# The FsrA‐Mediated Iron‐Sparing Response Regulates the Biosynthesis of the Epipeptide EPE in *Bacillus subtilis*

**DOI:** 10.1111/mmi.70039

**Published:** 2025-12-17

**Authors:** Sarah Miercke, Rabea Ghandour, Kai Papenfort, Thorsten Mascher

**Affiliations:** ^1^ Chair of General Microbiology TUD University of Technology Dresden Dresden Germany; ^2^ Institute of Microbiology, Chair of General Microbiology Friedrich Schiller University Jena Jena Germany; ^3^ Microverse Cluster, Friedrich Schiller University Jena Jena Germany

**Keywords:** *Bacillus subtilis*, cannibalism, gene expression, iron, RNA, RNA‐processing, post‐transcriptional gene regulation

## Abstract

Under severe nutrient‐limiting conditions, *Bacillus subtilis* is able to form highly resilient endospores for survival. However, to avoid this irreversible process, it employs an adaptive strategy termed cannibalism, a form of programmed cell death, to outcompete siblings and delay sporulation. One of the three cannibalism toxins, the epipeptide EPE, is encoded by the *epeXEPAB* operon. The pre‐pro‐peptide EpeX undergoes post‐translational modification and processing to be secreted as the mature EPE toxin. While EPE production is tightly regulated at multiple levels, this study focuses on the post‐transcriptional control by the small regulatory RNA FsrA, which is transcriptionally regulated by the global iron response regulator Fur. Electrophoretic mobility shift assays and RNA structure probing revealed two binding sites of FsrA within the intergenic region between *epeX* and *epeE* flanking the annotated *epeX* terminator structure and potentially interfering with RNA stability and *epeXEP* expression. Reporter assays revealed decreased levels of EPE‐dependent stress response in the absence of FsrA, indicative of a positive FsrA effect on gene expression under iron‐limited conditions; in contrast to the normally inhibitory activity of FsrA. Together, our findings suggest that under iron starvation, FsrA promotes RNA processing and enables *epeE* translation, ultimately enhancing EPE production.

## Graphical abstract

Under iron‐limited conditions, FsrA base‐pairs with the intergenic region between *epeX* and *epeE*, enhancing *epeE* translation and triggering EPE production. Toxin‐mediated cell lysis releases nutrients, including iron, which can be taken up by surviving cells. This study provides the first evidence of FsrA functioning as a positive post‐transcriptional regulator in secondary metabolism, expanding its known role beyond its exclusively described repressive functions on iron‐consuming enzymes in primary metabolism.

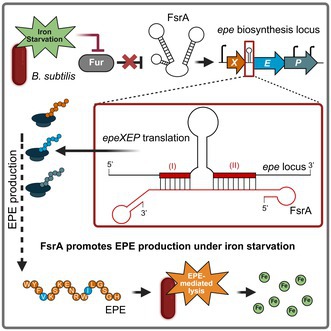

## 1 Introduction

For many bacteria living in fluctuating environments, survival depends on their ability to initiate adaptation processes that optimise resource allocation and population fitness by enabling subpopulations to transition between distinct physiological states in response to external stressors (Lopez et al. 2009). Especially under nutrient scarcity, some bacteria initiate complex developmental programs, which—in the case of the Gram‐positive model organism *B. subtilis* —involves motility, competence development, and ultimately sporulation. But prior to embarking on the irreversible and energy‐demanding formation of endospores, *B. subtilis* employs another strategy: cannibalism, a bacterial form of programmed cell death, to reclaim scarce resources, suppress or even eliminate competitors, and also to delay the formation of endospores, which is a strategy of last resort (González‐Pastor et al. 2003; González‐Pastor 2011).

*B. subtilis* produces three cannibalism toxins, the sporulation delay protein (SDP), the sporulation killing factor (SKF), and the more recently described epipeptide EPE. The latter belongs to a new class of ribosomally synthesised, post‐translationally modified, D‐amino acid‐containing peptides (González‐Pastor et al. 2003; Liu et al. 2010; Popp et al. 2020). EPE biosynthesis is encoded by the *epeXEPAB* operon, with *epeX* encoding the pre‐pro‐peptide, which is post‐translationally modified by the epimerase EpeE (Benjdia et al. 2017). The resulting pre‐peptide is subsequently processed by the membrane‐anchored peptidase EpeP in order to secrete the 17 amino acids linear mature toxin EPE (Popp et al. 2021). In addition, *epeAB* expresses an ATP‐binding cassette transporter that confers intrinsic autoimmunity against self‐produced toxin, which leads to slow and gradual dissipation of the membrane potential and pore formation (Popp et al. 2021; Butcher et al. 2007; Gebhard 2012). This ultimately results in subsequent cell lysis of the siblings and the release of valuable nutrients that feed the toxin producer (Popp et al. 2020; Popp et al. 2021).

While this controlled membrane disruption serves a cooperative function within the population, it simultaneously represents a threat to cellular integrity. Severe damage of the cell membrane, the outermost defensive barrier against external stressors, initiates the cell envelope stress response. This response involves the sensor kinase LiaS sensing the stress and triggering *liaIH* expression in a LiaR‐dependent manner (Butcher et al. 2007; Wolf et al. 2010). LiaIH forms a resistance determinant that protects against extracellular EPE toxicity due to reinforcement and stabilization of the cell membrane (Popp et al. 2021).

Although LiaIH contributes to membrane stabilisation against extracellular EPE, its expression is repressed by the transition state regulator AbrB (Popp et al. 2020; Jordan et al. 2007). This implies that cells remain unprotected if EPE is produced prematurely, underscoring the need for tight temporal control of toxin expression. Consequently, EPE production is synchronised with the transition to stationary phase, when activation of the master regulator of sporulation, Spo0A, downregulates AbrB and relieves repression of both the *liaI* and *epeX* promoters (Popp et al. 2021). This Spo0A‐dependent induction ensures that EPE biosynthesis occurs only under stress conditions, enabling the population to efficiently exploit nutrients released from lysed sibling cells.

Among these nutrients, iron is an essential trace element for bacteria and plays a critical role in various metabolic processes, including DNA synthesis, electron transport, respiration, as well as numerous enzymatic reactions within the tricarboxylic acid (TCA) cycle. However, elevated iron concentrations also have the potential to cause deleterious damage to various cellular components, such as DNA, macromolecules, and cell membranes, through the generation of reactive oxygen species (ROS) (Park et al. 2005; Imlay 2003). Hence, iron homeostasis must be tightly regulated both at the transcriptional and post‐transcriptional levels to control the expression of genes involved in iron acquisition, storage, export, and utilisation, and to minimise iron‐dependent oxidative stress (Pi and Helmann 2017).

One such mechanism is the iron‐sparing response, which prioritizes the conservation of iron for essential processes under iron‐limiting conditions. Central to this response is the small regulatory RNA FsrA, which is *trans*‐encoded and transcriptionally regulated by the global iron response regulator Fur (Gaballa et al. 2008). Upon Fur inactivation, FsrA is expressed and interacts post‐transcriptionally by base‐pairing with target messenger RNAs (mRNAs) encoding iron‐containing proteins. This interaction typically leads to mRNA destabilization or inhibition of translation (Gaballa et al. 2008). For example, FsrA downregulates low‐priority iron‐consuming enzymes such as the succinate dehydrogenase encoded by the *sdhCAB* operon and aconitase CitB, both involved in the TCA cycle (Pi and Helmann 2017; Smaldone, Antelmann, et al. 2012; Smaldone, Revelles, et al. 2012). By repressing these non‐essential iron‐dependent enzymes, FsrA contributes to the conservation of iron, likely making it available for enzymes and processes that are more critical under iron‐limiting conditions. This regulation enables the cell to adjust its metabolic priorities and respond efficiently to nutrient stress (Smaldone, Revelles, et al. 2012; Durand et al. 2021). While the influence of iron homeostasis on primary metabolism is well established, its role in modulating secondary metabolite biosynthesis, including toxin production, remains only poorly explored.

Notably, the activity of the radical S‐adenosyl‐L‐methionine (AdoMet) enzyme EpeE relies on an iron–sulphur ([4Fe‐4S]) cluster, which is essential for catalysing the conversion of two L‐amino acids into the D‐form within EpeX (Benjdia et al. 2017). Thus, EpeE function, and hence EPE production depends on the intracellular iron availability. Moreover, the *epe* locus was previously proposed as a potential target of FsrA, based on sequence similarities of the known interaction between RosA and FsrA (Durand et al. 2021). Further, in vivo crosslinking using 4′‐aminomethyl‐4,5′,8‐trimethylpsoralen (AMT), which was performed in M9 medium supplemented with 0.3% (w/v) glucose, identified *epeX* as a potential FsrA target, although this interaction has not yet been experimentally verified (Durand et al. 2021).

Therefore, the primary aim of this work was to unravel the potential interplay between the FsrA‐mediated iron‐sparing response and EPE production to further resolve the complex regulatory network of cell differentiation, resource management, and survival in *B. subtilis* . Accordingly, the role of FsrA in regulating EPE production was investigated, employing both in vitro and in vivo approaches to assess its impact on EPE biosynthesis and the EPE‐mediated cell envelope stress response under iron‐limited conditions.

## 2 Results

### 2.1 The *epeX*_IGR_*epeXE*_ RNA Is a Target of the Regulatory sRNA FsrA

Since FsrA‐binding sites were bioinformatically predicted using IntaRNA (Busch et al. 2008; Mann et al. 2017; Wright et al. 2014), we first aimed at investigating the potential regulatory connection between FsrA and *epeE* expression by defining the specific RNA region within the *epe* that is targeted by FsrA. Based on previously published transcriptome data derived from comprehensive tiling array analyses (Figure S1) (Dérozier et al. 2021; Nicolas et al. 2012), a pronounced growth condition‐dependent downshift in mRNA abundance was observed at the 3′‐end of *epeX*, leading to the assumption of post‐transcriptional regulation within this region. Therefore, an RNA fragment (207 nt) comprising the 3′‐end of the *epeX* mRNA and the intergenic region between *epeX* and *epeE* (IGR_*epeXE*_) was selected as a potential target for base pairing with FsrA (84 nt).

**FIGURE 1:**
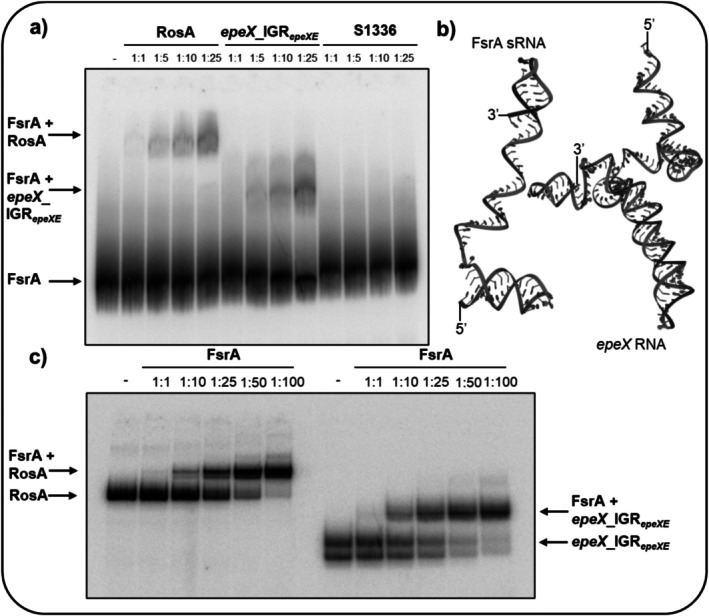
Interaction of the small regulatory FsrA and the *epeX*_IGR_*epeXE*_ RNA. (a) Electrophoretic mobility shift assay (EMSA) of 5′‐end radiolabelled FsrA with increasing molar ratios of unlabelled *epeX*_IGR_*epeXE*_ RNA. The complex formation of RosA and FsrA served as a positive control and S1336 was used as negative control RNA. Free FsrA as well as FsrA‐*epeX*_IGR_*epeXE*_ and FsrA‐RosA complex are indicated by arrows. (b) NuFold prediction of FsrA RNA (84 bp) and *epeX* RNA including sequence from +1 region till the terminator within IGR_*epeXE*_ (207 nt). (c) EMSA demonstrating the RNA–RNA complex formation of FsrA‐*epeX*_IGR_*epeXE*_ using 5′‐end radiolabelled *epeX*_IGR_*epeXE*_ RNA and increasing molar ratios of unlabelled FsrA. The FsrA‐RosA interaction was included as positive control.

The interaction between FsrA and this RNA fragment, *epeX*_IGR_*epeXE*_, was analyzed by electrophoretic mobility shift assays (EMSA). By maintaining a constant concentration of the 5′‐end radiolabeled FsrA and titrating increasing molar ratios of the *epeX_*IGR_*epeXE*_ RNA (ranging from 1:1 to 1:25), we observed a distinct shift at the 25‐fold molar ratio of *epeX_*IGR_*epeXE*_ to FsrA, indicating the formation of an RNA–RNA duplex. As a positive control, we confirmed the previously reported RosA‐FsrA interaction (Durand et al. 2021), which exhibited a characteristic shift comparable to the FsrA‐*epeX_*IGR_*epeXE*_ complex. The addition of S1336 RNA to the FsrA did not show any shift, and free FsrA could be detected only, confirming the specificity of the observed interaction (Figure 1a). Validation of the interactions was carried out by repeating the EMSA using 5′‐end radiolabeled *epeX_*IGR_*epeXE*_ and RosA as control. Increasing concentrations of FsrA were added to achieve molar ratios ranging from 1:1 to 1:100. The formation of a distinct shifted complex was detected for both RosA‐FsrA and FsrA‐*epeX_*IGR_*epeXE*_ by applying a molar ratio of 1:10, and a strong reduction of unbound labeled RNA was detected at a molar ratio of 1:100, supporting efficient binding between the two RNAs (Figure 1c).

Moreover, for the *epeX*_IGR_*epeXE*_ RNA, two bands were consistently detected under non‐denaturing conditions typically used for EMSAs (Vogt et al. 2024), likely reflecting secondary structure formation (Figure 1c). Although the RNA was denatured prior to loading, refolding and base‐pairing can readily occur during electrophoresis. In addition, complementary sequences within the *epeX* RNA (Figure 3b) suggest that the upper band may correspond to an RNA dimer formed through intermolecular pairing (Figure 1c).

The tertiary structures of the full‐length FsrA (84 nt) and *epeX* (207 nt) transcripts were predicted by the end‐to‐end deep network architecture of Nufold, which provides insights into the structural properties of RNAs (Figure 1b, Figure S2) (Kagaya et al. 2025). While at least one large helix motif is positioned between the +80 and +170 region of the *epeX* transcript, two distinct helical motifs located at the 5′‐end and 3′‐ends of FsrA enclose an unpaired single‐stranded region of the FsrA, which could serve as a potential target of RNA–RNA interactions by complementary base‐pairing (Figure S2).

### 2.2 IGR_*epeXE*_ Base‐Pairs With the Single‐Stranded Region of FsrA

**FIGURE 2:**
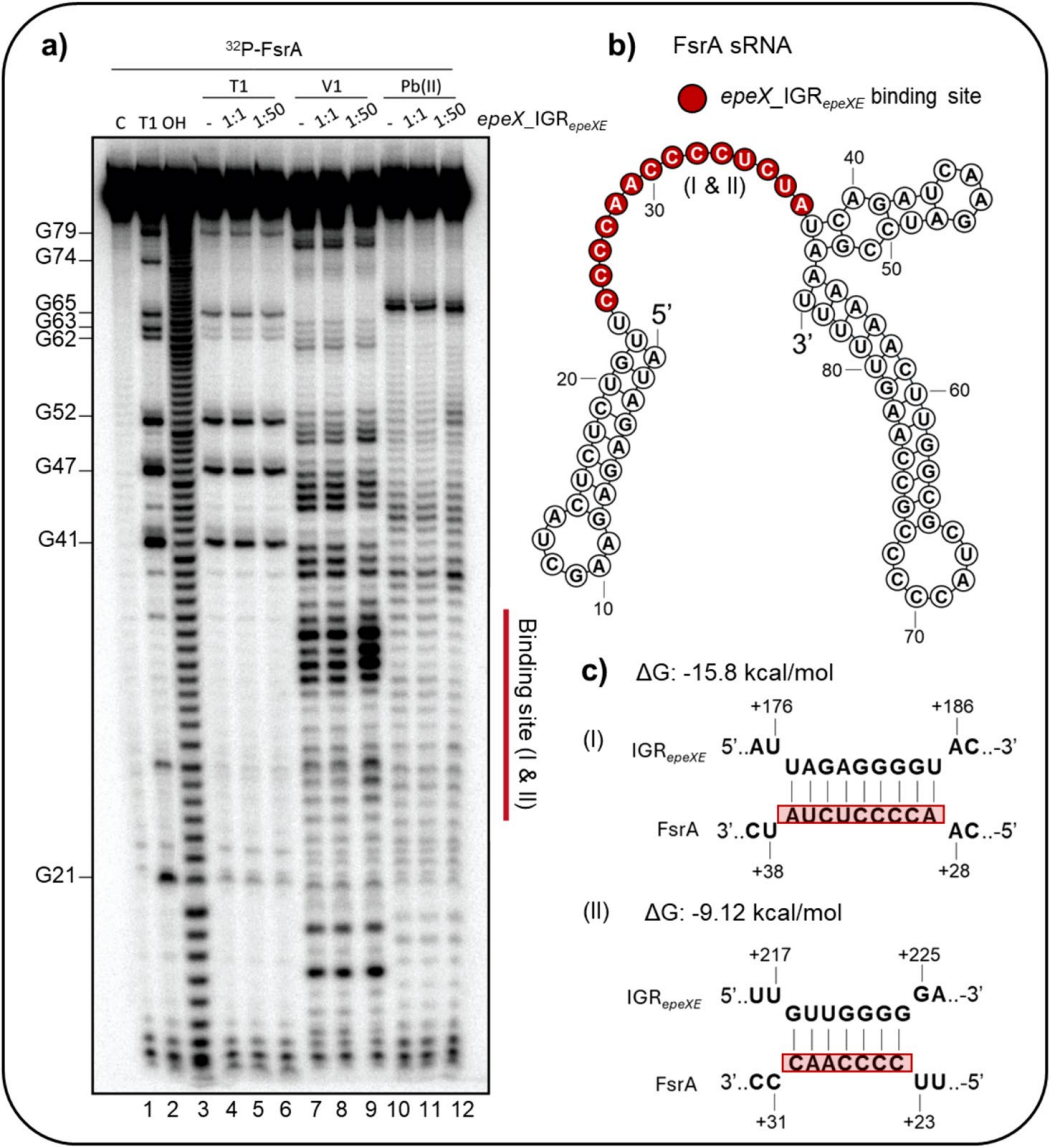
*epeX*_IGR_*epeXE*_ RNA base‐pairs with FsrA within a single‐stranded region. (a) in vitro structure probing of the 5′ end‐labelled FsrA with a length of 84 nt (0.4 pmol) was performed using RNase T1 (lanes 4–6), RNase V1 (lanes 7–9), and lead(II) acetate (lanes 10–12) in the presence and absence of *epeX*_IGR_*epeXE*_ RNA (0, 0.4 pmol, 20 pmol, respectively). Individual nucleotides and G‐residues were mapped by RNase T1 and alkaline (OH) treatment indicated relative to the FsrA transcription start site. (b) The secondary structure of FsrA was predicted by RNAfold web server and the *epeX*_IGR_*epeXE*_ binding sites were highlighted in red. (c) Sequential motif of *epeX*_IGR_*epeXE*_ binding sites on FsrA (red boxes) with free energies of −15.8 kcal/mol and −9.12 kcal/mol respectively are shown.

To analyse the secondary structure of FsrA and identify regions involved in target binding, structure probing was performed using RNase T1, RNase V1, and lead(II) acetate. RNase T1 and lead(II) acetate provided single‐nucleotide resolution by cleaving unpaired guanine (G) residues and flexible regions, respectively. RNase V1 was employed to probe base‐paired or structured regions of the RNA (Figure 2a). Notably, incubation of FsrA with 20 pmol of the *epeX*_IGR_*epeXE*_ RNA led to enhanced cleavage by RNase V1 within a region spanning nucleotides G21–G41 (Figure 2b).

This increased V1 sensitivity suggests that the previously unstructured region adopts a more double‐stranded conformation upon RNA–RNA interaction, consistent with predictions from secondary structure models in the absence of binding. Interestingly, this segment lacks guanine residues, making it invisible to T1 cleavage. However, cytosine (C) residues are rather abundant, and the ‐CCCCA‐, as well as ‐CCCCU‐ motifs are predicted to base pair with the *epeX*_IGR_*epeXE*_ RNA (Figure 2c) (Mann et al. 2017; Wright et al. 2014; Lorenz et al. 2011). While there is no absolute threshold, interactions with free energies more negative than approximately −10 kcal/mol are commonly considered stable enough to occur under physiological conditions (Tieng et al. 2023). These values suggest that the observed interactions are likely biologically relevant and may contribute to the post‐transcriptional regulation of *epeE* by FsrA.

### 2.3 The FsrA Base‐Pairs With Two Distinct Target Sites at the *epeX*_IGR_*epeXE*_ Transcript

**FIGURE 3:**
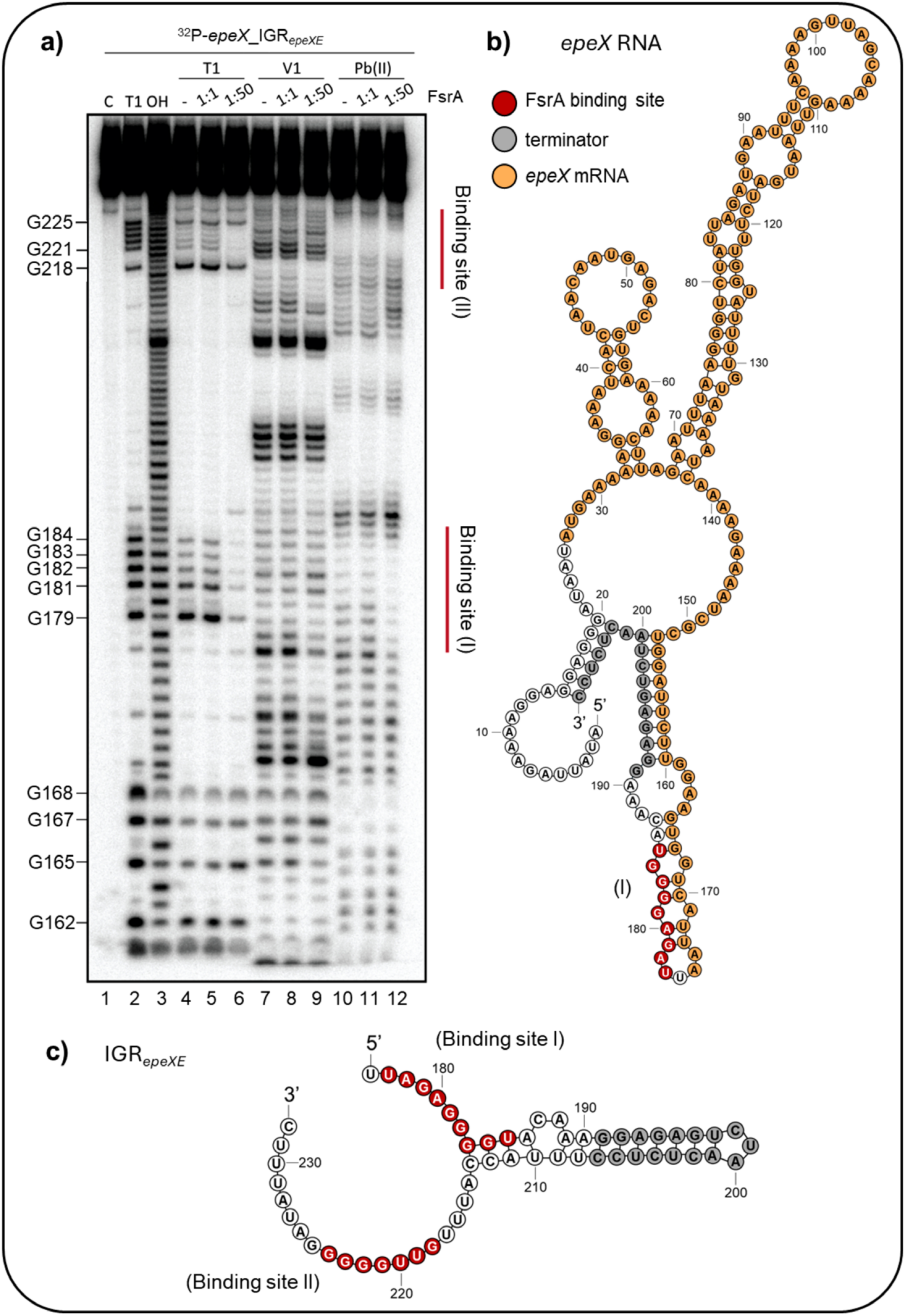
FsrA base‐pairs at two binding sites in the IGR between *epeX* and *epeE*. (a) in vitro structure probing of the 5′ end‐labelled *epeX*_IGR_*epeXE*_ RNA with a length of 83 nt covering the entire IGR_*epeXE*_ and 26 nt of the 3′ *epeX* coding sequence (0.4 pmol) was performed using RNase T1 (lanes 4–6), RNase V1 (lanes 7–9), and lead(II) acetate (lanes 10–12) in the presence of increasing amounts of FsrA (0, 0.4 pmol, 20 pmol, respectively). Individual nucleotides and G‐residues were mapped by RNase T1 and alkaline (OH) treatment indicated relative to the *epeX* transcription start site. The potential binding sites for FsrA are indicated by red lines. (b) The secondary structure of the full *epeX* transcript from transcription start site and including the terminator was determined by RNAfold prediction. The coding sequence of *epeX* (orange), terminator within the IGR_*epeXE*_ (grey) and FsrA binding site (red) as revealed in (a) are highlighted. (c) Structural prediction of the entire IGR_*epeXE*_ as target of both FsrA interactions.

We next aimed at unraveling the positions of FsrA binding to *epeX*_IGR_*epeXE*_. For that, RNA structure probing was performed using a 5′‐end labelled 83 nt *epeX*_IGR_*epeXE*_ RNA fragment, which encompasses the full IGR_*epeXE*_ (57 nt) and 26 nt of the 3′‐end of *epeX* mRNA sequence. Structure‐specific cleavage patterns were assessed following incubation with increasing concentrations of FsrA using RNase T1, RNase V1, and lead(II) acetate (Figure 3a). The presence of a 50‐fold molar excess of FsrA resulted in discernible alterations in cleavage sensitivity within two distinct regions, indicating specific RNA–RNA interactions. These regions, designated as binding sites (I) and (II), exhibited reduced cleavage upon treatment with RNase T1 in a concentration‐dependent manner, indicating FsrA‐mediated protection of previously accessible G‐residues or structural rearrangements that reduced their accessibility.

While the overall pattern of RNase V1 cleavage remained unchanged, the lead(II) acetate cleavage pattern was reduced in the presence of 20 pmol of FsrA, indicating a loss of flexible, single‐stranded regions upon complex formation. Mapping of the resolved binding sites onto the predicted secondary structure of the entire *epeX* transcript, covering the part from the transcription start site and including the terminator within the IGR_*epeXE*_, revealed the first FsrA binding site 5 nt upstream of the terminator structure (Figure 3b). Since the binding site (I) is located 1 nt downstream of the *epeX* coding sequence, prediction of the IGR_*epeXE*_ region only was performed, and both FsrA binding sites were mapped flanking the terminator structure (Figure 3c).

Bioinformatical predictions using IntaRNA corroborated these findings, disclosing two complementary sequences within FsrA that base‐pair with the respective regions at the IGR_*epeXE*_. Both contain the GGGGU motif, which is present in inverse order (Figure 2c). The presence of unpaired G‐residues within the FsrA binding sites within the IGR_*epeXE*_ was validated by RNA structure probing, as indicated by the RNase T1 sensitivity and a corresponding reduction in lead(II) acetate upon FsrA binding (Figure 3a). Taken together, our data support the presence of two FsrA binding sites responsible for FsrA‐mediated post‐transcriptional control of *epeXEP* expression.

### 2.4 Deletion of *fsrA* Results in Decreased EPE‐Dependent Stress Response

**FIGURE 4:**
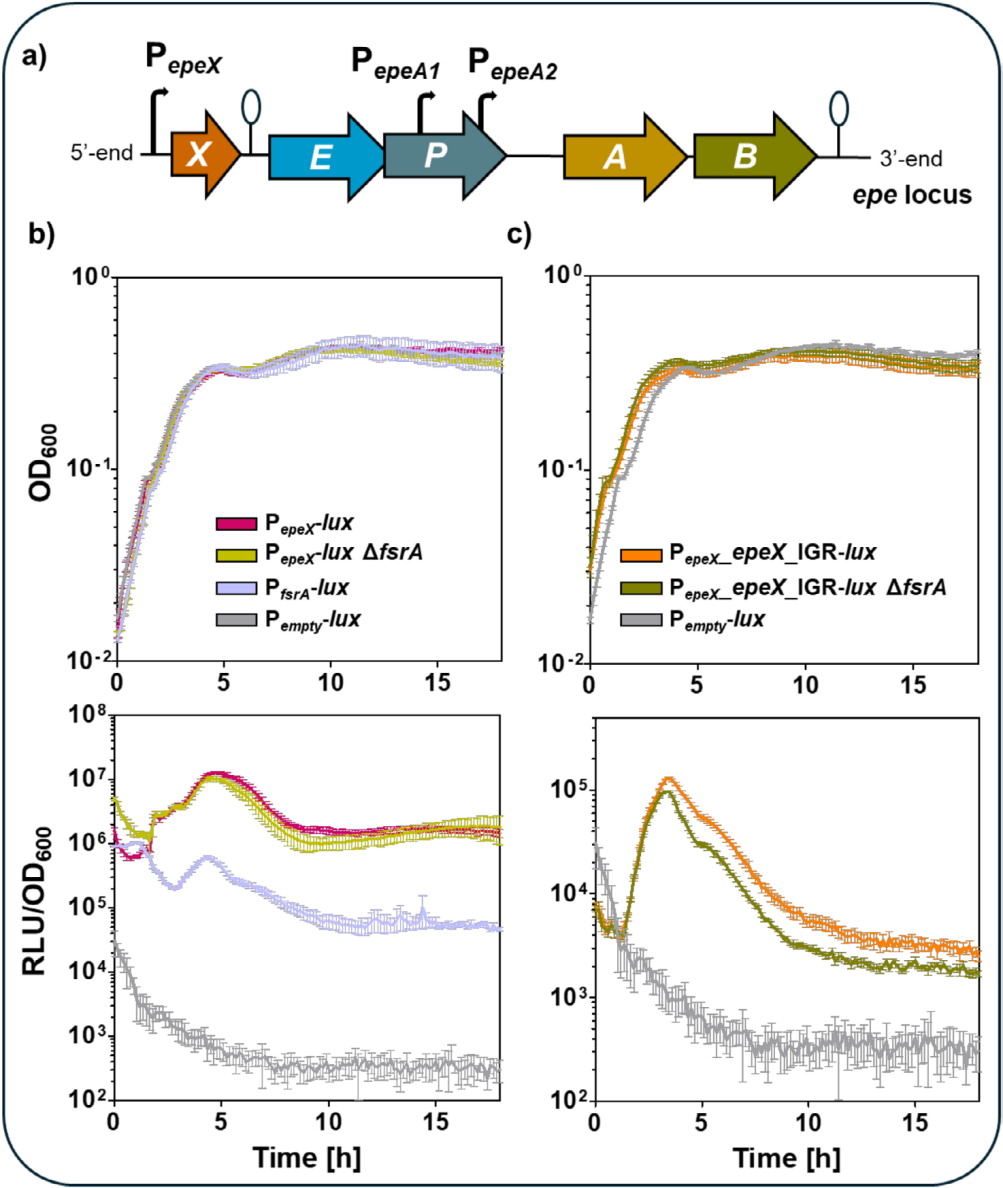
Deletion of *fsrA* decreases *epeEP* expression under iron starvation conditions. (a) The genetic organisation of the *epe* locus was depicted. (b, c) While the upper graphs show the growth curve as function of OD_600_ over time, the lower graphs visualise the RLU normalised to the corresponding OD_600_ values over time. The dynamic of (b) P_*epeX*_‐*lux* (pink) and (c) P_*epeX*__*epeX*_IGR‐*lux* (orange) activity in absence and presence of *fsrA* deletion (light and dark green, respectively) were displayed. The P_*fsrA*_‐*lux* (light purple) reporter was included as control, indicating the expression of FsrA under iron‐limited conditions. The assay was performed in adapted DSM medium without the addition of iron sulphate and supplemented with 20 μM EDDHA. The P_*empty*_‐*lux* reporter (light grey) further served as indicator of the background luminescence activity. The standard derivation as standard error of the mean was included as error bars to each time point of measurement. For simplification, genes deletions were highlighted by the delta symbol (Δ) and the precise genotype is listed in Table S1.

The demonstration of FsrA‐binding to the IGR_*epeXE*_ led to the question of the physiological relevance of this regulation under iron starvation. For that, reporter assays were performed to monitor the expression dynamics of EPE biosynthetic genes and the EPE‐mediated cell envelope stress response. A series of luciferase‐based reporter fusions was employed using the codon‐optimised *luxABCDE* (*lux*) cassette of *Photorhabdus luminescens* , which exhibits a half‐life of < 5 min, enabling real‐time monitoring of dynamic gene expression (Radeck et al. 2013; Francis et al. 2000). The transcriptional P_*epeX*_‐*lux* reporter was applied to monitor *epeX* expression. Additionally, the translational P_*epeX*_‐*epeX*‐IGR‐*lux* fusion, incorporating *epeX*, the intergenic region between *epeX* and *epeE* (IGR_*epeXE*_), as well as the native ribosome binding site of *epeE*, was constructed to assess translation of *epeE* and *epeP* (Figure 4a).

First, iron‐limited conditions were established by modifying the standard Difco sporulation medium (DSM) to examine the role of FsrA on EPE production in vivo. Specifically, the usual supplementation with iron sulphate was omitted, and the medium was further treated with the iron chelator ethylenediamine‐N,N′‐bis(2‐hydroxyphenylacetic acid) (EDDHA). The P_*fsrA*_‐*lux* reporter was used as an indicator of iron starvation to validate iron‐limited conditions. The reporter showed robust activity, peaking approximately 5 h after inoculation, confirming that the applied conditions were sufficient to activate the FsrA‐mediated iron‐sparing response (Figure 4b).

Next, the effect of iron limitation on *epeXEP* expression was assessed using both the transcriptional P_*epeX*_‐*lux* reporter and the translational P_*epeX*_‐*epeX*‐IGR‐*lux* fusion. P_*epeX*_ activity peaked at the onset of stationary phase and remained relatively high throughout growth, reaching its maximum approximately 5 h after inoculation (Figure 4b). This suggests that *epeX* is expressed continuously, with transcription levels increasing during the transition to stationary phase. The translational P_*epeX*_‐*epeX*‐IGR‐*lux* fusion was subsequently analyzed to gain further insights into the dynamics of *epeEP* expression. While its temporal expression profile closely resembled that of the P_*epeX*_‐*lux* reporter, the luminescence signal was approximately 100‐fold lower despite similar growth behavior (Figure 4c). This disparity likely reflects post‐transcriptional regulatory effects or reduced translation efficiency and supports the assumption that EPE biosynthesis initiates at the onset of stationary phase, whereas *epeX* expression occurs more constitutively. Notably, under iron‐depleted conditions, both reporters displayed expression dynamics comparable to those observed in standard DSM without modification (Figure S3a,b), indicating that the modified medium supported the induction of both *fsrA* and *epeXEP* expression (Figure 4, Figure S3).

Next, an *fsrA* deletion mutant was generated in the P_*epeX*_‐*lux* background to dissect the regulatory contribution of FsrA more directly. Reporter activity in this mutant remained comparable to the wild type strain, suggesting that P_*epeX*_ activity is not transcriptionally regulated by FsrA (Figure 4b, Figure S3a). Since the identified FsrA‐binding sites are located within the IGR_*epeXE*_, we next analysed *epeEP* expression using the P_*epeX*_‐*epeX*‐IGR‐*lux* reporter in the absence of FsrA. While the *fsrA* mutant did not exhibit any significant growth defects, the activity of the translational P_*epeX*_‐*epeX*‐IGR‐*lux* fusion was approximately two‐fold reduced from the onset of the stationary phase onwards (Figure 4c, Figure S4). Notably, this effect was not visible under iron‐replete conditions (Figure S3b). These findings support a post‐transcriptional role of FsrA in promoting *epeEP* expression, potentially through stabilisation of the transcript or enhanced translation efficiency, thus facilitating EPE production under iron‐limiting conditions.

**FIGURE 5:**
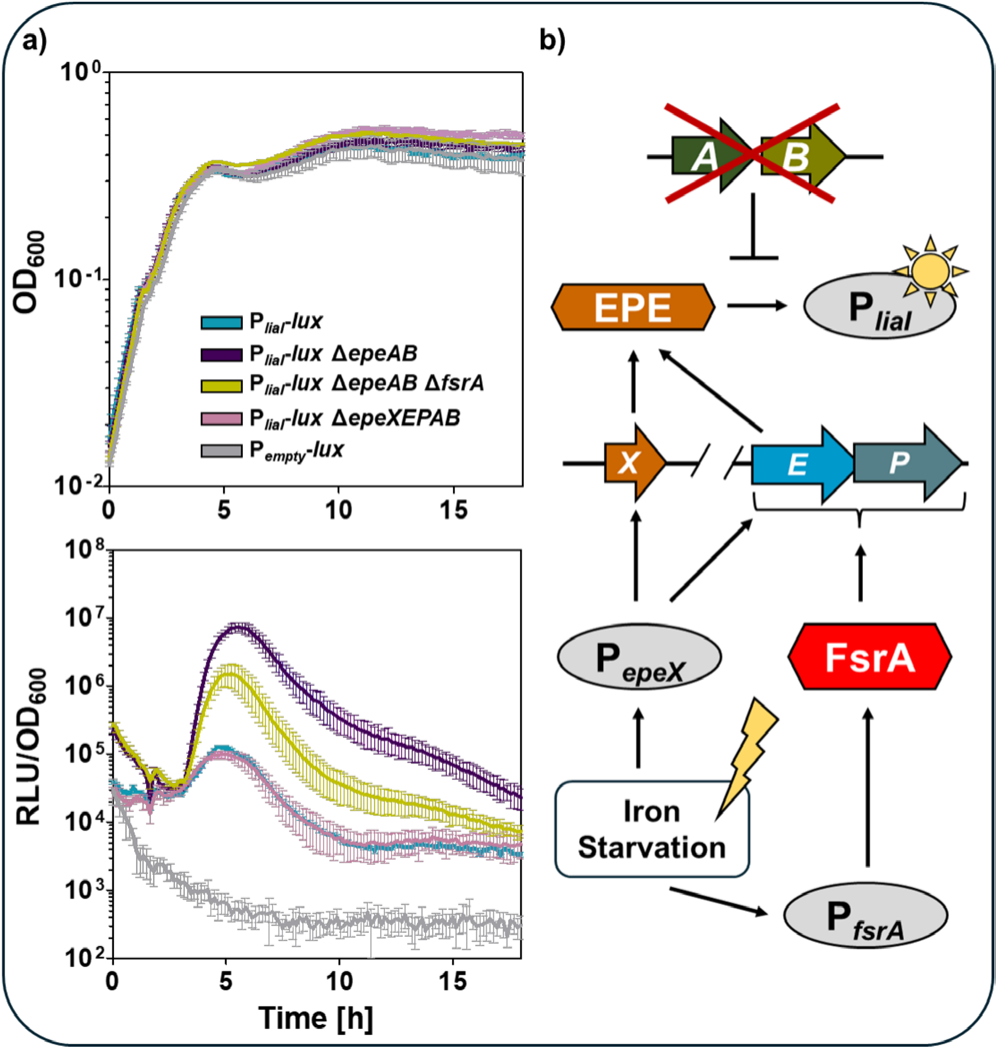
The EPE‐dependent stress response is affected by the FsrA. (a) The upper graph shows the growth curve as function of OD_600_ over time, the lower graph visualises the RLU normalised to the corresponding OD_600_ values over time. The dynamic of P_*liaI*_‐*lux* activity in absence and presence of gene deletion was displayed. The P_*empty*_‐*lux* reporter served as indicator of the background luminescence activity. The analysis was performed in adapted DSM medium without iron sulphate and supplemented with 20 μM EDDHA. The standard derivation as standard error of the mean of the biological and technical triplicates was included as error bars to each time point of measurement. For simplification, genes deletions were highlighted by the delta symbol (Δ) and the precise genotype is listed in Table S1. (b) Schematic overview of the regulation of EPE biosynthesis under iron‐limited conditions. Under conditions of iron starvation, both P_*fsrA*_ and P_*epeX*_ are active due to the loss of repression by their respective inhibitors, Fur and AbrB. Activation of P_*epeX*_ drives the transcription of the *epeXEP* operon. In parallel, P_*fsrA*_ promotes the expression of the FsrA, which in turn enhances *epeEP* expression through post‐transcriptional regulation. This regulatory cascade results in the production of mature EPE toxin, which subsequently induces P_*liaI*_ activity as part of the cell envelope stress response. Deletion of the toxin immunity genes *epeAB* increases the sensitivity of the P_*liaI*_‐*lux* reporter strain to EPE‐mediated membrane damage.

As EPE specifically activates the Lia‐system, the P_*liaI*_‐*lux* reporter was applied to monitor the dynamics of EPE‐induced stress (Figure 5a). When combined with an *epeAB* deletion, this reporter provides a sensitive readout of the membrane‐damaging action of mature, extracellular EPE. In the absence of the toxin autoimmunity transporter EpeAB, the reporter becomes approximately 100‐fold more sensitive to EPE‐mediated cell envelope stress response. P_*liaI*_ activity peaked around 5.5 h in both wild type and Δ*epeAB* backgrounds (Figure 5a). The timing closely correlates with the previously observed onset of *epeXEP* expression, which occurs slightly earlier (Figure 4a,b). The temporal alignment between toxin production and stress response activation suggests that the Lia‐system responds rapidly to the presence of EPE. These findings indicate that both EPE biosynthesis and the resulting membrane stress are initiated at the transition to stationary phase, supporting the conclusion that EPE exerts its biological effect immediately upon its secretion.

Next, the P_*liaI*_‐*lux* reporter in an *epeAB* deletion background was employed to evaluate the physiological impact of FsrA on EPE‐mediated stress response. Deletion of *fsrA* led to an approximately 10‐fold reduction in P_*liaI*_ activity compared to the P_*liaI*_‐*lux* Δ*epeAB* reporter strain, although EPE activity was not entirely abolished, as evidenced by comparison with the *epeXEPAB* mutant (Figure 5a). However, this reduction in P_*liaI*_‐*lux* activity was not detectable under iron‐replete conditions (Figure S3c), indicating that the FsrA‐dependent contribution to EPE‐mediated stress is specifically manifested under iron limitation.

Together, these results indicate that under iron limitation, both P_*fsrA*_ and P_*epeX*_ are activated due to the loss of repression by Fur and AbrB, respectively. P_*epeX*_ activation drives transcription of the *epeXEP* operon, while P_*fsrA*_ induces *fsrA* expression (Figure 5b). FsrA, in turn, enhances *epeEP* expression post‐transcriptionally, promoting the production of mature EPE toxin, which activates the P_*liaI*_‐mediated cell envelope stress response. The substantial decrease in P_*liaI*_ activity upon *fsrA* deletion indicates that FsrA is required for efficient EPE production under iron‐limited conditions (Figure 5b). To date, positive regulatory effects of FsrA on gene expression have not been documented, highlighting a potentially novel mode of action for this conserved iron‐sparing response sRNA.

## 3 Discussion

Iron limitation poses a significant challenge for bacteria and triggers global metabolic changes to ensure survival and balance resource allocation (Pi and Helmann 2017). This response requires tight regulation at the transcriptional and post‐transcriptional levels to fine‐tune and rapidly adapt gene expression in response to environmental stressors. In *B. subtilis* , iron homeostasis is primarily regulated by the ferric uptake regulator Fur, which senses intracellular iron levels (Pi and Helmann 2017; Gaballa et al. 2008; Pinochet‐Barros and Helmann 2020). Under conditions of sufficient iron availability, Fur represses genes involved in iron acquisition and transport, as well as the iron‐sparing response sRNA FsrA (Pi and Helmann 2017; Smaldone, Antelmann, et al. 2012). Under iron‐limited conditions, FsrA, the central mediator of the iron‐sparing response in *B. subtilis* , is active and has so far exclusively been described as an inhibitor of iron‐dependent enzymes (Smaldone, Antelmann, et al. 2012; Smaldone, Revelles, et al. 2012). The results in this study provide the first evidence of a positive regulatory role for FsrA, since its activity contributes to the post‐transcriptional control of EPE biosynthesis under iron‐limited conditions, presumably to release iron from the sibling cells by EPE‐mediated cell lysis.

The sRNA‐mediated iron‐sparing response is conserved across numerous bacterial species, including *Shigella flexneri* , *Vibrio cholerae* , and *E. coli* (Massé et al. 2007). In these organisms, prioritization of iron usage is coordinated by RyhB, a Fur‐regulated sRNA (Massé et al. 2005). RyhB operates with the RNA chaperone Hfq, which stabilizes sRNA‐mRNA interactions and recruits RNase E for target degradation (Panja and Woodson 2012). In contrast, *B. subtilis* FsrA (84 nt) functions independently of Hfq but requires partial support from three small basic proteins, namely FbpA, FbpB, and FbpC (Gaballa et al. 2008). Their expression is also transcriptionally regulated by Fur, and FbpABC are proposed to act as RNA chaperones supporting FsrA‐mediated regulation (Gaballa et al. 2008). FbpB was also shown to enhance FsrA repression of the *lutABC* operon, illustrating its role in facilitating effective sRNA‐mRNA interactions (Smaldone, Antelmann, et al. 2012). Moreover, FbpB has been proposed to facilitate recruitment of RNases to FsrA targets, analogous to Hfq‐dependent recruitment of the RNA degradosome in *E. coli* , but the exact mechanism of FbpABC function remains to be elucidated (Smaldone, Antelmann, et al. 2012). Whether FbpB alone or in combination with FbpA and FbpC contributes to FsrA‐mediated regulation of the *epe* locus has not yet been investigated but represents an interesting direction for future studies.

However, it is conceivable that RNA chaperons may be less critical for the FsrA‐*epeX*_IGR interaction, as the base‐pairing involves highly stable GC‐rich regions. Mechanistically, FsrA contains C‐rich regions comparable to the RNA sponge RoxS, which is conserved in Bacilli and Staphylococci (Durand et al. 2021). RoxS facilitates base‐pairing with G‐rich target sequences, such as ribosome binding sites, despite imperfect complementarity (Durand et al. 2021). In this context, the presence of four consecutive GC base pairs, occurring in both FsrA binding motifs within the IGR_*epeXE*_, has been suggested to be critical for effective sRNA‐mRNA interaction, which commonly impacts mRNA stability and translation initiation (Durand et al. 2021).

Similarly, the canonical FsrA‐mediated iron sparing response typically involves repression of translation and enhanced mRNA degradation, for example, via RBS sequestration and RNA destabilisation (Smaldone, Antelmann, et al. 2012; Durand et al. 2021). Previously identified FsrA targets include *gltD*, *sdhC*, *citB*, *gltAB*, *leuABCD*, and *lutABC*, most of which encode proteins involved in primary metabolism, particularly the TCA cycle, a central pathway for energy production and biosynthetic precursor generation, e. g., citrate, succinate, and malate (Gaballa et al. 2008; Smaldone, Antelmann, et al. 2012; Smaldone, Revelles, et al. 2012; Durand et al. 2021). Contrary to the typical inhibitory function of FsrA in gene expression, a positive mode of action, in which the FsrA is required for EPE biosynthesis, was demonstrated (Figures 4 and 5). This is the first example of an FsrA‐mediated, post‐transcriptional activation of genes involved in secondary metabolism, specifically toxin maturation. Interestingly, the *E. coli* equivalent, sRNA RyhB, is primarily known to downregulate iron‐dependent enzymes, but has also been reported to activate translation initiation of target genes (Massé et al. 2007; Massé et al. 2005). For instance, RyhB promotes translation of *shiA* under iron‐limited conditions, by base‐pairing with its 5′‐UTR. RyhB binding disrupts an intrinsic inhibitory secondary structure, otherwise occluding the RBS, by conformational changes (Prévost et al. 2007).

**FIGURE 6:**
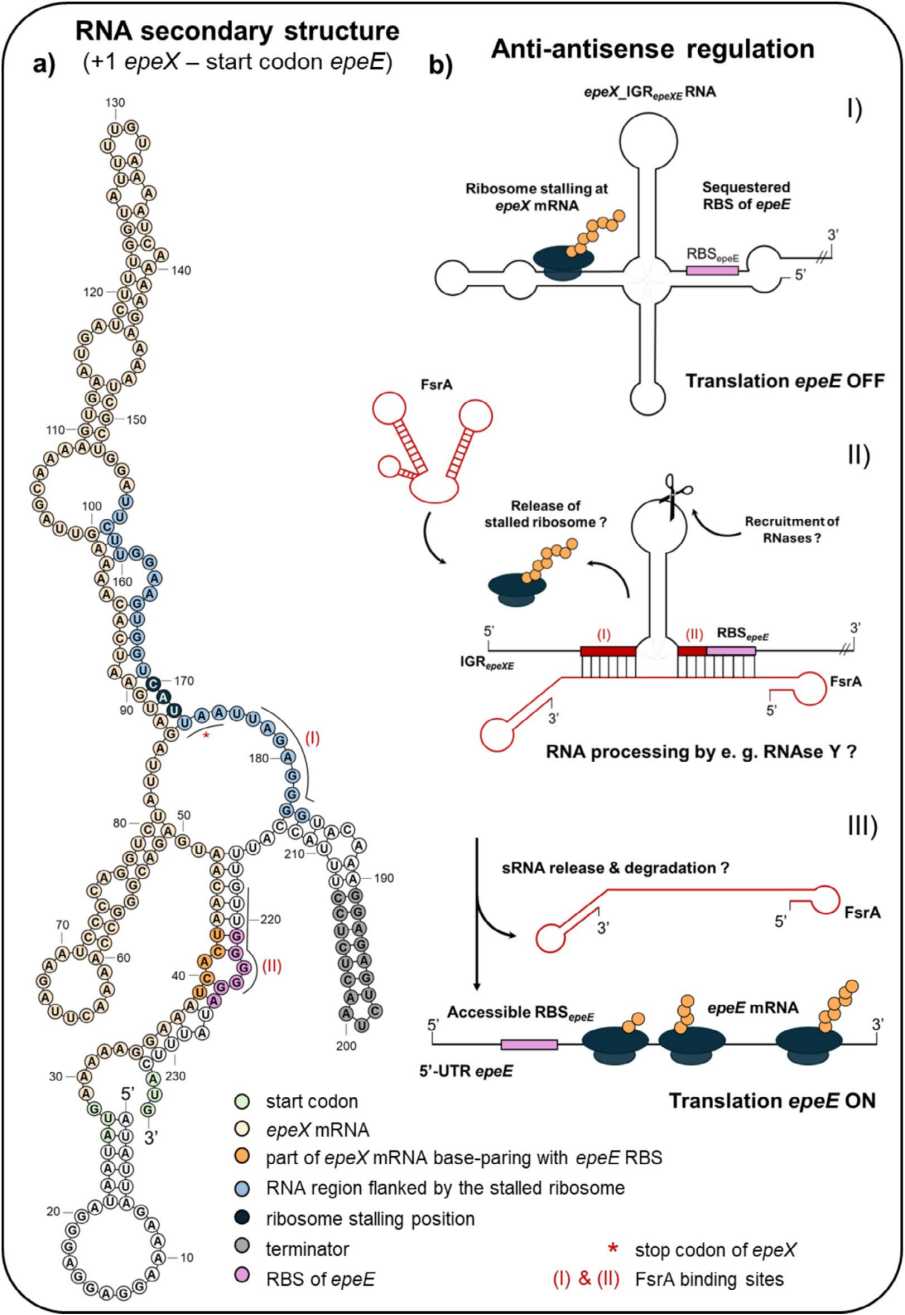
Hypothetical mechanism of FsrA‐mediated activation of *epeE* expression. (a) Bioinformatic prediction of the secondary RNA structure spanning the region from *epeX* transcription start site till *epeE* start codon. Distinct features within the structure were highlighted: start codon (light green), *epeX* mRNA (light yellow), the fifth and sixth coding triplet of the *epeX* mRNA base‐pairing with the RBS of *epeE* (orange), RNA region flanked by the stalled ribosome (light blue), ribosome stalling position (A‐site) (dark blue), terminator (grey), stop codon (red asterisk), and FsrA binding site (red latin numbers). (b) Hypothetical anti‐antisense mechanism of FsrA‐dependent *epeE* regulation. (I) In the absence of FsrA, the 5′‐end of the *epeX* mRNA base‐pairs with the RBS of *epeE*, thereby inhibiting *epeE* translation. Ribosome stalling occurs at the last coding triplet of the *epeX* mRNA, preventing complete translation of *epeX* and potentially stabilising the transcript. (II) Under iron starvation conditions, FsrA base‐pairs at two positions within the IGR_*epeXE*_ region, overlapping with the 3′‐UTR of *epeX* and the RBS of *epeE*. This interaction ensures full translation of *epeX*. FsrA presumably recruits RNase Y, leading to processing of the *epeX*_IGR_*epeXE*_ RNA and subsequent FsrA release and degradation. (III) Following RNA processing and sRNA degradation, the RBS of *epeE* becomes accessible, allowing translation initiation and *epeE* expression.

Bioinformatic predictions of the secondary structure of the transcript spanning the region from *epeX* transcription start site to the *epeE* start codon revealed a similar structure, in which the RBS of *epeE* is sequestered through base‐pairing with the fifth and sixth coding triplets of the *epeX* mRNA. This proposed intrinsic secondary structure could inhibit translation initiation of *epeE* by preventing ribosome access to its RBS (Figure 6a,b(I)). The secondary structure may also contribute to the exceptional stability of the *epeX* mRNA, which has a half‐life exceeding 15 min, in contrast to the less stable *epeEP* transcript (Hambraeus et al. 2003). Moreover, a recent study demonstrated that the *epeX* transcript exhibits the highest ribosome occupancy and pausing in *B. subtilis* , with the last coding triplet (CAT) of the *epeX* mRNA acting as a ribosome stall site (Han et al. 2025). The ribosome at this position occupies a region spanning 14 nt upstream and 12 nt downstream of the ribosome A site (Han et al. 2025), thereby potentially impeding full translation of *epeX*. Interestingly, FsrA base‐pairs with two G‐rich regions within the IGR_*epeXE*_, flanking the rho‐independent transcription terminator (Mandell et al. 2022). These binding sites overlap both the 12‐nucleotide downstream region occupied by the stalled ribosome and the RBS of *epeE*, respectively (Figure 6a). Through this positioning, FsrA may modulate local RNA secondary structure and influence ribosome accessibility.

Such sRNA‐mRNA interactions are often coupled to the recruitment of ribonucleases, which mediate targeted mRNA degradation or processing to fine‐tune transcript abundance and translation efficiency (Papenfort and Melamed 2023). In *B. subtilis* , the major endoribonuclease is RNase Y, a functional analogue of RNase E in *E. coli* (Trinquier et al. 2020). RNase Y is a 59 kDa enzyme belonging to the histidine‐aspartate domain (HD) superfamily of metal‐dependent phosphohydrolases, requiring magnesium ions for catalytic activity (Lehnik‐Habrink, Newman, et al. 2011). RNase Y is anchored to the cell membrane via an N‐terminal transmembrane helix, and its membrane localisation is critical for proper function (Laalami et al. 2024). RNase Y plays a crucial role in mRNA decay, processing of polycistronic RNAs, and regulation of S‐box riboswitches. The cleavage specificity is often enhanced through interactions with the RicA‐RicF‐RicT complex (Y complex) (Trinquier et al. 2020; DeLoughery et al. 2018; Shahbabian et al. 2009; Dubnau et al. 2023). Notably, the *epe* locus has been identified as one of the RNase Y targets, with *epeE* mRNA abundance decreasing between 3‐ and 16‐fold upon RNase Y depletion or delocalisation via cytoplasmic expression (Laalami et al. 2024; Lehnik‐Habrink, Schaffer, et al. 2011).

However, the observed decrease in *epeE* transcript abundance upon RNase Y depletion is not readily explained by the canonical role of RNase Y in mRNA decay (Condon 2003). While loss of RNase Y activity would typically lead to transcript stabilization and increased RNA levels, reduced transcript abundance has been repeatedly observed. Such downregulation is often attributed to indirect regulatory effects, for instance through altered expression of transcriptional repressors or global regulators, as previously described for the *eps* and *tapA‐sipW‐tasA* operons, where increased *sinR* mRNA stability upon RNase Y depletion led to increased repression of these target genes (Laalami et al. 2024; Lehnik‐Habrink, Schaffer, et al. 2011; Laalami et al. 2013; Taggart et al. 2025).

Nevertheless, direct processing‐dependent stabilisation has also been described, such as for the *lip* gene encoding an extracellular lipase, where RNase Y cleavage generates a more stable monocistronic *lip* mRNA species (Lehnik‐Habrink, Schaffer, et al. 2011). Comparable phenomena are well documented for RNase E in *E. coli* , which can stabilise specific transcript isoforms or facilitate sRNA‐mediated regulation through processing within polycistronic operons, for example, for the *glmS*‐*glmU* operon, leading to the stable monocistronic *glmS* (Joanny et al. 2007; Kalamorz et al. 2007). Moreover, *glmS* expression is positively regulated by the sRNA GlmZ, which potentially recruits RNase E for processing and/or stabilises the monocistronic *glmS* transcript by structural rearrangement (Kalamorz et al. 2007). By analogy, the FsrA‐IGR_*epeXE*_ sRNA‐mRNA interaction may contribute to *epeE* regulation by promoting RNase Y‐dependent processing within the IGR_*epeXE*_ that likewise generates a stable monocistronic *epeE* transcript. This provides a potential mechanistic explanation for its differential regulation and for the observed decrease in *epeE* abundance upon RNase Y depletion or delocalisation (Laalami et al. 2024; Lehnik‐Habrink, Schaffer, et al. 2011).

In general, examples of sRNAs acting as positive regulators of gene expression are scarce in bacteria. Among the few well‐characterised cases in *E. coli* are RhyB, the homolog of FsrA (Prévost et al. 2007), GadY, which stabilises *gadX* mRNA, and DsrA (Opdyke et al. 2004), which positively affects translation by altering inhibitory hairpin formation in the *rpoS* mRNA (Repoila et al. 2003). All of these sRNAs depend on the RNA chaperone Hfq to facilitate their regulatory function. In *Vibrio cholerae* , the Qrr sRNAs have also been reported to exert both positive and negative regulation of target genes (Hammer and Bassler 2007).

Taken together, these examples underscore the rarity of sRNA‐mediated activation, highlighting FsrA as an important and versatile regulatory RNA in *B. subtilis* with a potentially unique function. While the precise molecular mechanism by which FsrA promotes *epeE* expression remains to be fully elucidated, it is tempting to speculate that FsrA may act via an anti‐antisense mechanism, in which FsrA base‐pairs at two positions within the structured IGR_*epeXE*_ region to enhance transcript stability and/or translation. Moreover, AlphaFold modelling (Abramson et al. 2024) suggests that the stem‐loop architecture within the IGR_*epeXE*_ remains preserved upon binding (Figure S5); this dual interaction may facilitate *epeE* translation by modulating RNA accessibility and promoting RNase Y processing, potentially coupled to subsequent FsrA degradation (Figure 6b (I–III)).

While the precise mechanism remains to be elucidated, this study provides new insights into the FsrA‐mediated regulation of EPE biosynthesis. The production of EPE under iron‐limited conditions might serve as a physiological advantage due to toxin‐mediated cell lysis accompanied by the release of nutrients, such as iron, to maintain survival. Both the epipeptide EPE and the iron‐sparing response sRNA FsrA are conserved among Firmicutes, and FsrA homologs have been identified across a wide range of bacterial species, including Bacilli, Staphylococci, and Listeria. This broad conservation suggests that the coupling of iron homeostasis to toxin‐mediated differentiation could represent a more general adaptive strategy among Gram‐positive bacteria. Whether this specific regulatory interaction between FsrA and the *epe* locus is conserved in other organisms remains to be investigated, but it represents an intriguing direction for future studies. In addition, further experiments, such as site‐directed mutagenesis and allele‐specific suppression, could provide more direct evidence for the specific base‐pairing sites involved and clarify the functional relevance of each FsrA binding site, representing an important avenue for future work.

Although the present study focused primarily on the regulation of EPE biosynthesis, FsrA likely regulates additional targets, including other enzymes that utilize iron–sulphur clusters as cofactors. According to *Subtiwiki* (Elfmann et al. 2025) a comprehensive list of all known iron‐dependent enzymes is provided in the Supporting Information and potential FsrA interactions were predicted via IntaRNA (Busch et al. 2008; Mann et al. 2017; Wright et al. 2014; Raden et al. 2018). The majority of the [4Fe‐4S] cluster enzymes, such as AddB, Fer, and SdhB, which are involved in DNA synthesis, electron transfer, and the TCA cycle, respectively (Alonso et al. 1993; Green et al. 2003; Hederstedt and Rutberg 1981) belong to the primary metabolism. In contrast, the enzymes AlbA, SkfB, and EpeE are known to be iron‐dependent members of the SAM radical enzyme superfamily and are part of the secondary metabolism, specifically the maturation of the peptide toxins (Benjdia et al. 2017; Zheng et al. 2000; Grell et al. 2018).

While this study highlights the regulation of the radical‐SAM epimerase EpeE (Benjdia et al. 2017), AlbA, and SkfB belong to the subfamily of radical SAM sactisynthases, which catalyze the thioether bond formation between the sulfur atom of a cysteine residue and *α*‐carbon of an acceptor residue in subtilosin A and SKF (Grell et al. 2018; Flühe et al. 2012). Potential FsrA binding sites, consisting of G‐rich sequences, could be predicted within the 5′‐UTR of both *albA* and *skfB*, suggesting additional FsrA‐mediated toxin regulation under iron starvation conditions (Supporting Information, Figure S6). Altogether, this study provides new insights into the dual regulatory roles of FsrA: not only as a repressor of primary metabolism but also potentially as an activator of secondary metabolism, thereby expanding our understanding of post‐transcriptional control mechanisms and the iron‐sparing response in *B. subtilis* .

## 4 Materials and Methods

### 4.1 Bacterial Strains and Growth Conditions

*B. subtilis* and *E. coli* were cultivated at 37°C and 220 rpm agitation in lysogeny broth (LB) [1% tryptone (w/v), 0.5% (w/v) yeast extract, and 1% (sodium chloride)]. For solidification of agar plates, 1.5% (w/v) agar‐agar was applied. Iron‐limited conditions were achieved by supplementing adapted DSM media [8 g/L nutrient broth, 1 g/L KCl, 1 mM MgSO_4_, 10 μM MnCl_2_, 500 μM CaCl_2_] with 20 μM ethylenediamine‐*N,N*′‐bis(2‐hydroxyphenylacetic acid) (EDDHA). All *B. subtilis* and *E. coli* strains used in this study were listed in Table S1. Selection of *B. subtilis* strains harboring a resistance cassette was carried out by applying chloramphenicol (5 μg mL^−1^), kanamycin (10 μg mL^−1^), spectinomycin (100 μg mL^−1^), phleomycin (2 μg mL^−1^), or for macrolide‐lincomycin‐streptogramin B (MLS) resistance, 1 μg mL^−1^ erythromycin combined with 25 μg mL^−1^ lincomycin. *E. coli* strains carrying plasmids were selected by using ampicillin (100 μg mL^−1^). Transformation of *B. subtilis* and *E. coli* was performed according to (Harwood and Cutting 1990; Sambrook and Russell 2001).

### 4.2 Cloning Proceedings

Chemicals and enzymes applied for standard cloning procedures including restriction digestion, ligation, and polymerase chain reaction (PCR) were obtained from NEB (New England Biolabs, Ipswich, USA) and carried out according to the manufacturer's recommendation. Reporter fusions were generated by applying the pBS3C*lux* vector (Radeck et al. 2013), which was digested with EcoRI and SalI and ligated with the same‐wise digested PCR product of the desired promoter fragment. PCR purifications and plasmid preparations were undertaken using the corresponding kits from Süd‐Laborbedarf GmbH (Gauting, DE). Obtained plasmids (Table S2) were verified by PCR amplification and sequencing. Oligonucleotides used are listed in Table S3.

### 4.3 T7 Transcription and 5′‐End‐Labelling of RNA

RNAs were generated through in vitro transcription as described previously (Ghandour et al. 2025). In brief, a DNA template containing the T7 promoter was amplified via PCR using designated primers (KPO‐10824/KPO‐10825 for FsrA, KPO‐10826/KPO‐10827 for RosA, and KPO‐10828/KPO‐10829 for *epeX*_IGR_*epeXE*_) and transcribed using the MEGAscript transcription kit (AM1334, Thermo Fisher, Waltham, USA). The resulting RNA (20 pmol) was purified and dephosphorylated with calf intestinal alkaline phosphatase (NEB, Ipswich, USA), followed by phenol:chloroform:isoamyl alcohol (P:C:I) extraction. The dephosphorylated RNA was then radiolabelled with [^32^P]‐γATP (25 μCi) by incubation with 1 unit of polynucleotide kinase (NEB, Ipswich, USA) at 37°C for 1 h. Purification was carried out using a denaturing 6% polyacrylamide/7 M urea gel, after which the RNA was eluted overnight at 4°C in RNA elution buffer (0.1 M sodium acetate, 0.1% SDS, 10 mM EDTA) and recovered via P:C:I extraction and ethanol precipitation.

### 4.4 Electrophoretic Mobility Shift Assay

RNA–RNA interactions between *epeX_*IGR_*epeXE*_ and FsrA were assessed using electrophoretic mobility shift assays (EMSA), as described previously (Vogt et al. 2024). Briefly, 5′‐end radiolabelled RNA was denatured and then mixed with 1 μg of yeast RNA (Invitrogen, #AM7118, Thermo Fisher, Waltham, USA) and 1X structure buffer (0.01 M Tris, pH 7, 0.1 M KCl, 0.01 M MgCl₂). Unlabelled RNA was subsequently added at different molar ratios, and the reaction mixture was incubated at 37°C for 15 min. Reactions were then halted by adding native loading buffer (50% glycerol, 0.5X TBE, 0.2% bromophenol blue) and immediately loaded onto a pre‐chilled 6% native polyacrylamide gel. After electrophoresis, gels were dried, and signals were visualized through autoradiography using a Typhoon imager (Amersham Biosciences, Amersham, UK).

### 4.5 RNA Structure Probing

RNA structure analysis and mapping of *epeX_*IGR_*epeXE*_‐FsrA interaction sites were conducted following previously established protocols (Ghandour et al. 2025). In summary, 0.4 pmol of RNA labelled at the 5′‐end was first denatured and then rapidly cooled on ice. Subsequently, 1 μg of yeast RNA (Invitrogen, AM7118, Thermo Fisher, Waltham, USA) and 1X structure buffer (0.01 M Tris, pH 7, 0.1 M KCl, 0.01 M MgCl₂) were added. Unlabelled RNA was then introduced in varying molar ratios, followed by incubation at 37°C for 10 min. The samples were subjected to enzymatic or chemical cleavage using either RNase T1 (0.1 U; Ambion, AM2283, Thermo Fisher, Waltham, USA) for 3 min, lead(II) acetate (final concentration: 6.25 mM; Sigma‐Aldrich, 316512‐5G) for 1 min and 45 s, or RNase V1 (2 × 10^−5^ U; Ambion, AM2275, Thermo Fisher, Waltham, USA) for 1 min. The reactions were halted by adding an equal volume of GLII loading buffer. For sequencing ladder generation, 0.8 pmol of 5′ end‐labelled RNA was treated with RNase T1 or subjected to alkaline (OH) hydrolysis, and the reactions were stopped using GLII loading buffer (94% Formamide, 18 mM EDTA, 0.025% SDS, 0.025% bromophenol blue, 0.025% xylene cyanole). All samples were resolved via denaturing polyacrylamide gel electrophoresis (PAGE) on a 12% polyacrylamide gel containing 7 M urea. Gels were dried and signals were detected by autoradiography using a Typhoon imager (Amersham Biosciences, Amersham, UK).

### 4.6 Luciferase‐Based Reporter Assay

Luciferase activity of strains carrying the corresponding reporter fusions was assayed as previously described (Radeck et al. 2013). In brief, cultures were grown overnight in LB media with appropriate antibiotics, and day cultures were inoculated 1:100 in adapted DSM media without antibiotics, supplemented with 10 μM EDDHA. Cultures were grown until OD_600_ 0.2–0.4. Subsequently, the cells were diluted to an OD_600_ of 0.05 in fresh media, and 100 μL cell suspension were transferred into a 96‐well plate (black wells, clear bottom, Greiner Bio‐One, Frickenhausen, Germany). Following this, the 96‐well plate was placed into the Synergy Neo 2 Plate reader (BioTek, Winooski, USA), which is under the control of the Gen5 software (BioTek, Winooski, USA). The samples were incubated at 37°C, and the OD_600_ as well as the relative luminescence units (RLU) were monitored every 9 min for 18 h. The assays were performed in at least biological and technical triplicates.

### 4.7 Bioinformatic Tools

Secondary structures of RNAs were predicted using the RNAfold web server (http://rna.tbi.univie.ac.at/cgi‐bin/RNAWebSuite/RNAfold.cgi) (Gruber et al. 2008). The RNA base‐pairing interactions were predicted by applying IntaRNA (https://rna.informatik.uni‐freiburg.de) (Busch et al. 2008; Mann et al. 2017; Wright et al. 2014), and tertiary structures of RNAs were predicted with NuFold (https://colab.research.google.com/github/kiharalab/nufold/blob/master/ColabNuFold.ipynb) (Kagaya et al. 2025). Expression profiles based on comprehensive tiling array data were observed from Genoscapist (https://genoscapist.migale.inrae.fr/) (Dérozier et al. 2021; Nicolas et al. 2012). AlphaFold (https://alphafoldserver.com/) was applied to generate the model of the FsrA‐*epeX* interaction (Abramson et al. 2024).

## Author Contributions

**Sarah Miercke:** conceptualisation, experimental work, data analysis and interpretation, writing – original draft. **Rabea Ghandour:** experimental work, data analysis and interpretation, writing – review and editing. **Kai Papenfort:** writing – review and editing. **Thorsten Mascher:** conceptualisation, writing – review and editing.

## Funding

This work was supported by Deutsche Forschungsgemeinschaft, 503931087, 390713860, 504017689. European Research Council, 101088027.

## Conflicts of Interest

The authors declare no conflicts of interest.

## Supporting information


**Data S1:** mmi70039‐sup‐0001‐DataS1.xlsx.

**Data S2:** mmi70039‐sup‐0002‐DataS2.pdf.

## Data Availability

The data that support the findings of this study are available on request from the corresponding author.
