# Supplementary material for: The FsrA‐Mediated Iron‐Sparing Response Regulates the Biosynthesis of the Epipeptide EPE in Bacillus subtilis
Source: Mol Microbiol. 2025 Dec 17;125(2):108–22. doi: 10.1111/mmi.70039 (PMC12871923; doi:10.1111/mmi.70039)
Supplement: Supplementary file 2 — Data S2: mmi70039‐sup‐0002‐DataS2.pdf. [file MMI-125-108-s001.pdf]

Supplementary Information

The FsrA-Mediated Iron-sparing Response Regulates the Biosynthesis of the Epipeptide EPE in *Bacillus subtilis*

FsrA Regulates EPE Biosynthesis in *Bacillus subtilis*

Sarah Miercke[1], Rabea Ghandour[2], Kai Papenfort[2,3], and Thorsten Mascher[1]

Sarah.miercke@tu-dresden.de (+49 35146334511)

Rabea.ghandour@uni-jena.de (+49 3641949317)

Kai.papenfort@uni-jena.de (+49 3641949311)

Thorsten.mascher@tu-dresden.de (+49 35146340420)

[1] TUD University of Technology Dresden, Chair of General Microbiology, Dresden, Germany

[2] Friedrich Schiller University Jena, Institute of Microbiology, Chair of General Microbiology, Jena, Germany

[3] Microverse Cluster, Friedrich Schiller University Jena, Jena, Germany

Keywords: RNA-Processing, Post-Transcriptional Gene Regulation; Iron; Gene Expression; RNA; Cannibalism; *Bacillus subtilis*

Table of Contents

Table S1: Bacterial strains applied in this study.
Table S2: Plasmids used in this study.
Table S3: DNA oligonucleotides used in this study.

Figure S1: Expression profile of the *epe* locus.
Figure S2: Predicted local distance difference test (pLDDT) for RNA structure predictions using Nufold.
Figure S3: Impact of FsrA on *epeXEP* expression and EPE-mediated stress under iron-replete conditions.
Figure S4: Maximum activity of the P_{liaI}-*lux* Δ*epeAB* and P_{epeX}_*epeX*_IGR-*lux* reporter strains in absence and presence of the *fsrA*.
Figure S5: AlphaFold model of the FsrA-*epeX* interaction.
Figure S6: Predicted FsrA binding sites at the 5'-end of *albA*, and *skfB*.

Supplementary Information:

Table S1: Bacterial strains applied in this study.

Strain	Genotype*	Reference
DH10*β*	laboratory wild type strains	Durfee et al. 2008 [1]
W168	*trpC2*	Laboratory stock
3A38	*B. subtilis* NCIB3610 *comI* (Q12L) = DK1042	Konkol et al. 2013 [2]
TMB6217	3A38 *sacA*::P_{liaI}-*lux_cat*, *epeAB*::*spec*	This study
TMB6136	3A38 *sacA*::P_{liaI}-*lux_cat*	This study
TMB6350	3A38 *sacA*::P_{empty}-*lux_cat*	This study
TMB6247	3A38 *sacA*::P_{liaI}-*lux_cat*, *epeAB*::*spec epeX*::*mls*	This study
TMB7140	3A38 *sacA*::P_{liaI}-*lux_cat*, *epeXEPAB*::*spec*	This study
TMB6170	3A38 *sacA*::P_{epeX}-*lux_cat*	This study
TMB6865	3A38 *sacA*::$P_{epeX\_}$*epeX*_IGR-*lux_cat*	This study
TMB6832	3A38 *sacA*::P_{epeX}-*lux_cat fsrA*::*kan*	This study
TMB6891	3A38 *sacA*::$P_{epeX\_}$*epeX*_IGR-*lux_cat fsrA*::*kan*	This study
TMB6753	3A38 *sacA*::P_{liaI}-*lux_cat*, *epeAB*::*spec fsrA*::*kan*	This study
TMB7323	3A38 *sacA*::P_{FsrA}-*lux_cat*	This study

**cat*: chloramphenicol resistance; *kan*: kanamycin resistance; *spec*: spectinomycin; *mls*: macrolide, licosamide, and streptogramin B resistance; *lux*: *luxABCDE*

Table S2: Plasmids used in this study.

Plasmid/vector	Genotype*	Reference
pBS3C*lux*	*sacA*' … '*sacA luxABCDE*, *bla*, *cat*	Radeck et al. 2013 [3]
pBS3C*αlux*	*sacA*' … '*sacA*, *luxABCDE*, *bla*, *cat* (exchange-able RBS-site of *luxA*), *lacZα*	Popp et al. 2017 [4]
pBS3C*lux*_P_{fsrA}	*sacA*' P_{fsrA}-*luxABCDE_cat* '*sacA*	This study
pBS3C*lux*_P_{liaI}	*sacA*' P_{liaI}-*luxABCDE_cat* '*sacA*	This study
pBS3C*lux*_P_{epeX}	*sacA*' P_{epeX}-*luxABCDE_cat* '*sacA*	This study
pBS3C*lux*_P_{epeX}-*epeX*-IGR	*sacA*' P_{epeX}-*epeX*-IGR-*luxABCDE_cat* '*sacA*	This study
pBS3C*lux*_P_{empty}	*sacA*' *luxABCDE_cat* '*sacA*	This study

**cat*: chloramphenicol resistance; *bla*: ampicillin resistance

Table S3: DNA oligonucleotides used in this study.

Name	Sequence 5' to 3'	Description
KPO-10824	gttttttttaatacgactcactatag ATAGAGAGAAGCTACTCTCTGTTCC	*In vitro* transcription of FsrA
KPO-10825	TAAAAAACTTGGCGGGGGTAGC	
KPO-10826	gttttttttaatacgactcactatag GAAAGCGCCTACACTTACCTATTTC	*In vitro* transcription of RosA
KPO-10827	AAAAAAAGCCTGGCTCAGCAGAC	
KPO-10828	gttttttttaatacgactcactatag GCTGGATTCTTGGAAGTGGTC	*In vitro* transcription of *epeX*-IGR$_{epeXE}$ IGR
KPO-10829	GAAATATCCCCCAACAAATGGT	
TM8458	atgcgaattcGAGCAGGACGGACTGATTTAAC	P_{fsrA} amplification
TM8459	agtcgtcgacGAACAGAGAGTAGCTTCTCTC	
TM8031	GAAGAGCTGACAGATTGCAGC	Verification of *fsrA* deletion
TM8032	GGGAGTCATGCTGGATAGAGG	
TM7686	agtctctagaTCCCTCCTCCTTTTCTAATATAAAC	P_{epeX} amplification
TM8135	agtcggtctcgaattGGACCTTTATATGATAGTACCGC	
TM7986	acgtcgacCAACAAATGGTAAAGGAGAG	P_{epeX}-*epeX*-IGR amplification
TM8135	agtcggtctcgaattGGACCTTTATATGATAGTACCGC	
TM2262	GAGCGTAGCGAAAAATCC	Verification of reporter fusions
TM2263	GAAATGATGCTCCAGTAACC	

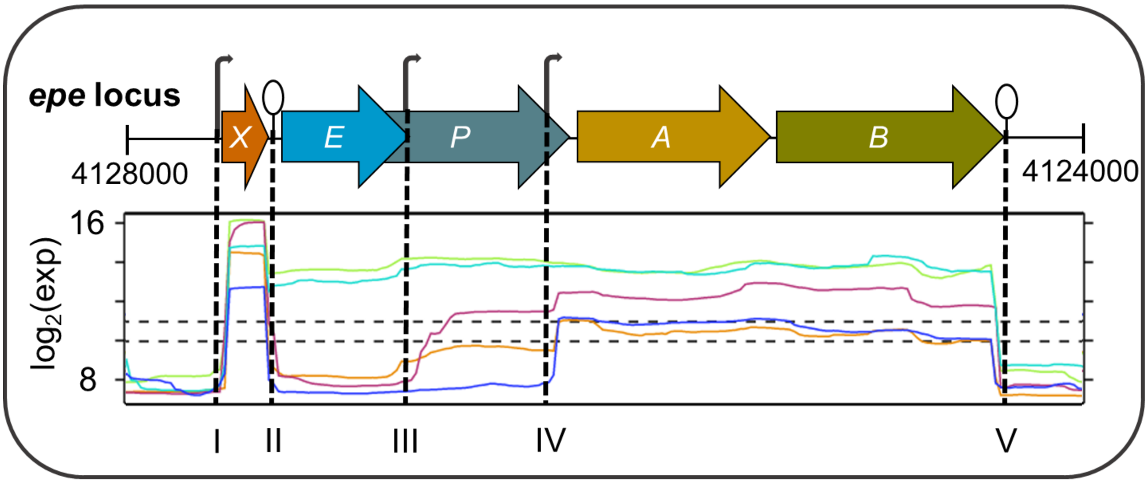

Figure S1: Expression profile of the *epe* locus.

Mapping of mRNA abundance based on comprehensive tiling array data indicates 3 upshifts of mRNA abundance. While the first coincides with the *epeX* promoter **(I)**, the following two correspond to the already mapped promoters located within e*peP* ($P_{epeA1/2}$) **(III, IV)**. Moreover, a strong growth condition-dependent downshift at the 3'-end of *epeX* was reported by analysing over 200 different growth conditions **(II)**. Conditions: mid-log growth in M9 minimal medium with malate (turquois), shift from CH minimal medium to SM sporulation medium 2 h (light green) and 6 h (orange, blue) after shift, biofilm growth after 36 h in MSgg medium at 30 °C (red). In contrast to the growth-independent termination at the 3'-end of *epeB* **(V)**, growth-dependent changes in particular *epeEP* mRNA abundance cannot exclusively explained by the mapped terminator **(II)** and raise the hypothesis of post-transcriptional regulation at the 3'-end of *epeX*. The expression profile was derived from *Genoscapist* [5],[6]

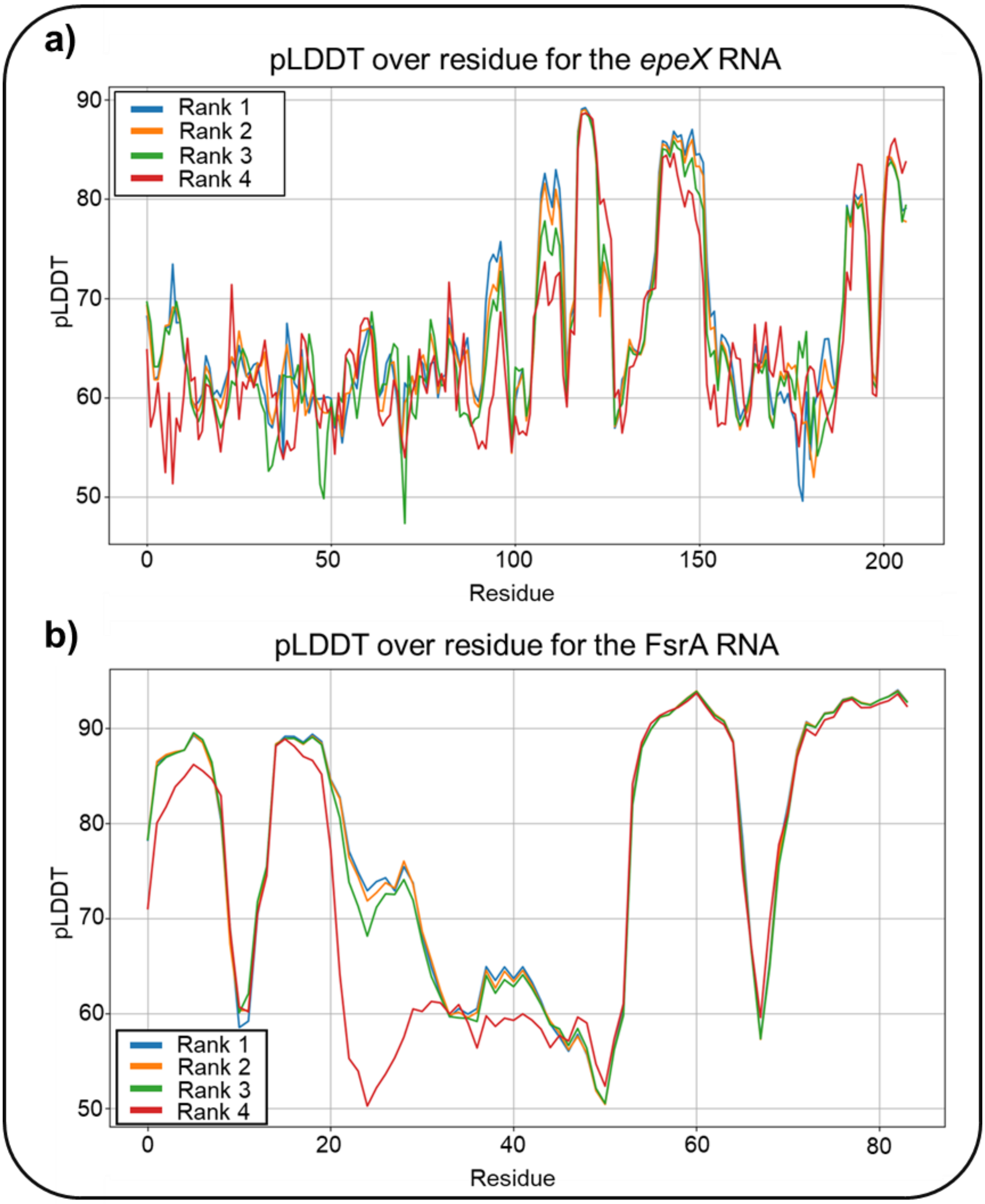

Figure S2: Predicted local distance difference test (pLDDT) for RNA structure predictions using Nufold.

a) pLDDT scores across residues of the *epeX* transcript. **b)** Predicted pLDDT scores across residues of the FsrA transcript. Predictions were generated using Nufold [7], and four ranked structural models are shown (Ranks 1–4), each represented by a different colour. Higher pLDDT values indicate greater confidence in the predicted local structure.

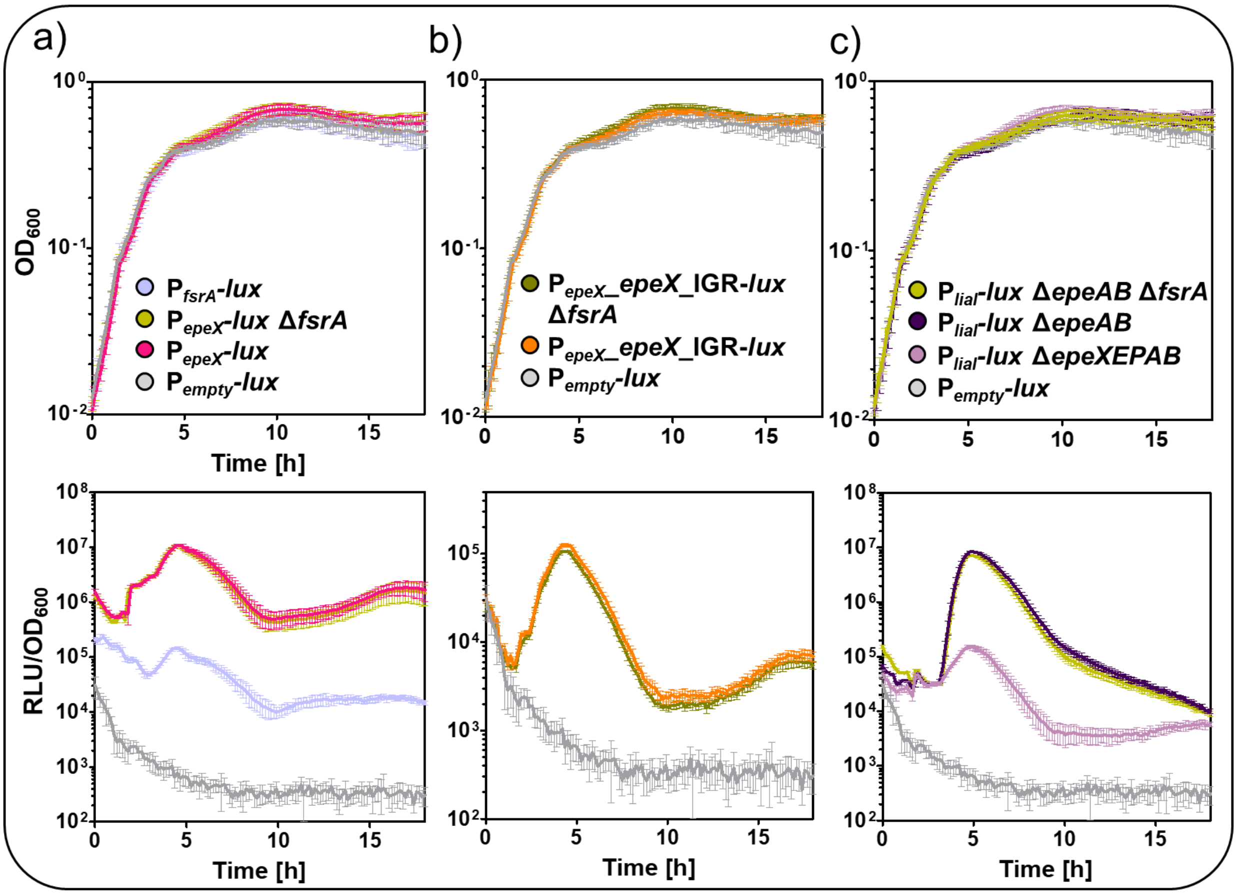

Figure S3: Impact of FsrA on *epeXEP* expression and EPE-mediated stress under iron-replete conditions.

a-c) While the upper graphs show the growth curve as function of OD_{600} over time, the lower graphs visualise the RLU normalised to the corresponding OD_{600} values over time. The dynamic of a) P_{fsrA}-*lux* (light purple), P_{epeX}-*lux* (pink), **b)** P_{epeX}_*epeX*_IGR-*lux* (orange), and P_{liaI}-*lux* Δ*epeAB* (dark purple) activity in absence and presence of fsrA deletion were displayed. The assay was performed in standard DSM medium (iron-replete conditions). The P_{empty}-*lux* reporter (light grey) further served as indicator of the background luminescence activity. The SEM was included as error bars to each time point of measurement. For simplification, genes deletions were highlighted by the delta symbol (Δ) and the precise genotype is listed in Table S1.

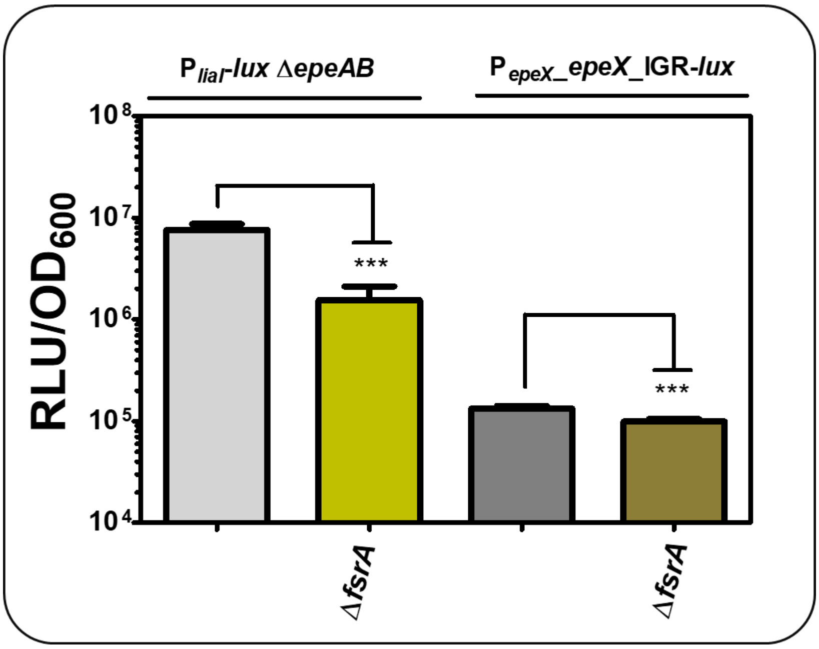

Figure S4: Maximum activity of the P*liaI*-*lux* Δ*epeAB* and P*epeX*_*epeX*_IGR-*lux* reporter strains in absence and presence of the *fsrA*.

Maximum RLU/OD_{600} values over time were shown as bar graphs and the standard derivation of biological and technical triplicates was included as error bars. Statistical significance as result of t-test performed as two sample assuming with unequal variances was indicated by: ns = not significant; * = p<0.05; ** = p<0.01; *** = p<0.001.

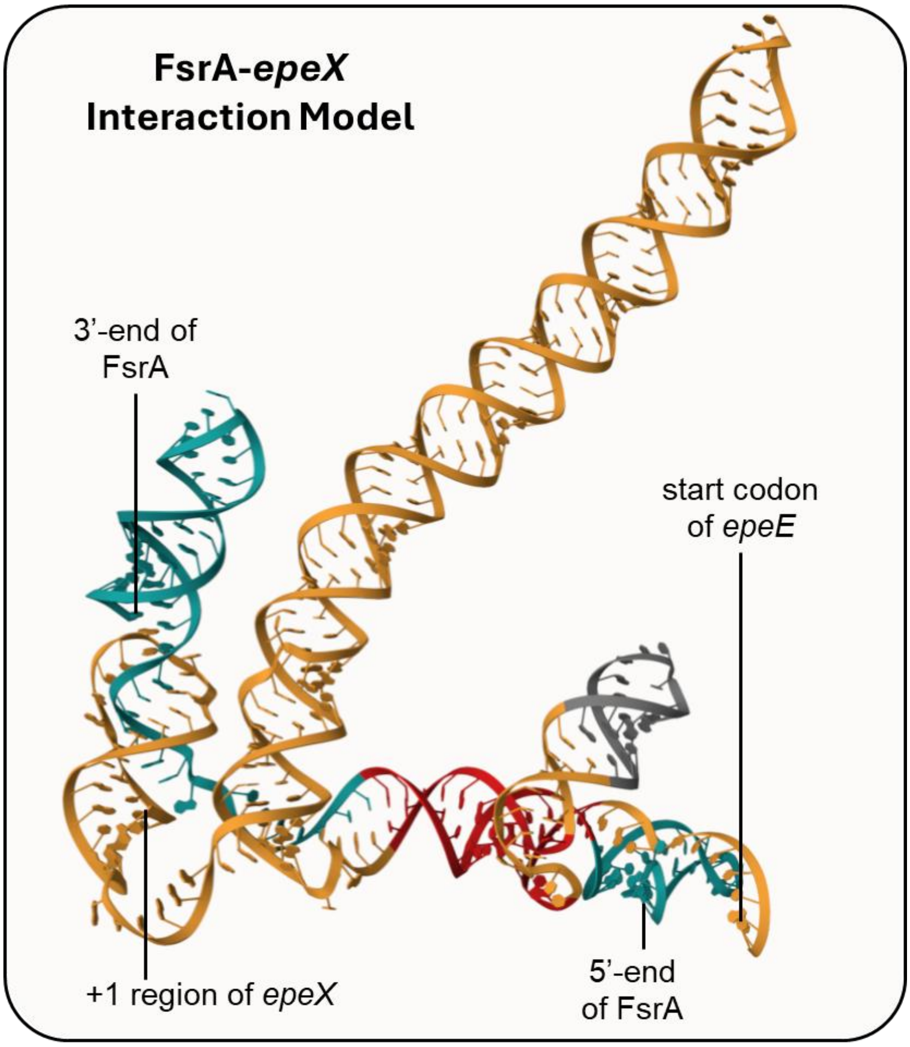

Figure S5: AlphaFold model of the FsrA-*epeX* interaction.

The predicted [8] complex between FsrA (green) and the *epeX* RNA region spanning from the +1 site of *epeX* to the *epeE* start codon (orange) is shown. FsrA binding sites are highlighted in red, while the intrinsic terminator sequence is indicated in grey. The applied RNA boundaries are marked by the corresponding start and end positions. The model suggests that FsrA interacts with two distinct sites flanking the central stem–loop structure, which remains preserved upon binding.

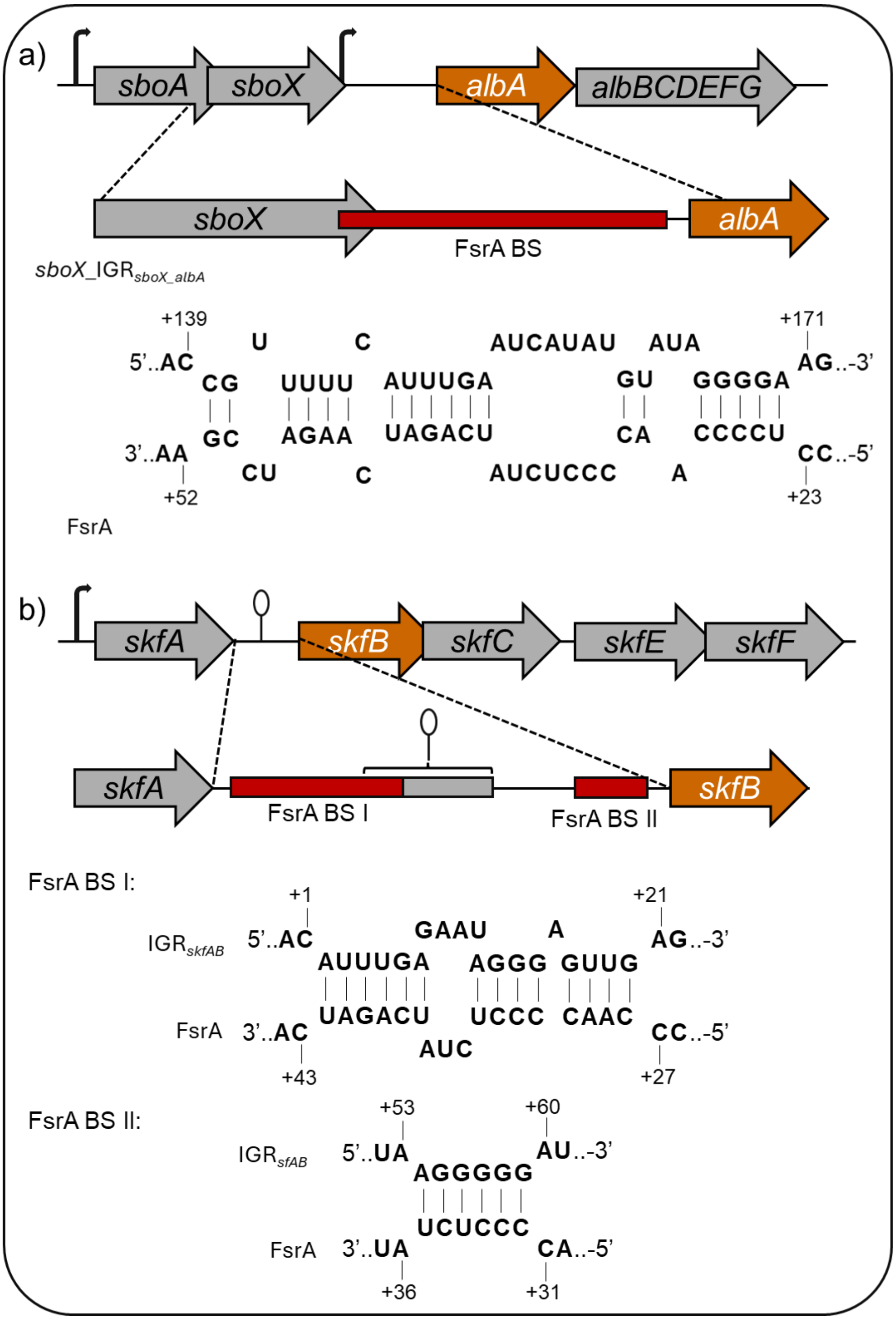

Figure S6: Predicted FsrA binding sites at the 5'-end of *albA*, and *skfB*.

Potential FsrA binding sites (BS) were predicted using IntaRNA [9]–[11]. **a)** Prediction revealed a binding site for FsrA at the 5'-end of *albA*, partially overlapping with the 3'-end of *sboX* and the IGR between *sboX* and *albA*. The genetic organisation is shown, with the predicted FsrA binding site highlighted in red. The precise base-pairing interactions between FsrA and its target sequence is depicted below. The gene encoding the iron-dependent enzyme AlbA is highlighted in orange. **b)** Two FsrA FsrA binding sites were predicted within the IGR between *skfA* and *skfB*. These binding sites, marked in red, overlap with the rho-independent terminator hairpin or the RBS of *skfB*. The corresponding sequence motifs are displayed below the genetic organisation, and *skfB*, encoding another iron-dependent enzyme, is also highlighted in orange.

References for Supplementary Information

[1] Durfee, T. *et al.* The Complete Genome Sequence of *Escherichia coli* DH10B: Insights into the Biology of a Laboratory Workhorse ▿ †. *Journal of bacteriology* 190, 2597–2606; 10.1128/JB.01695-07 (2008).

[2] Konkol, M. A., Blair, K. M. & Kearns, D. B. Plasmid-encoded ComI inhibits competence in the ancestral 3610 strain of Bacillus subtilis. *Journal of bacteriology* 195, 4085–4093; 10.1128/JB.00696-13 (2013).

[3] Radeck, J. *et al.* The Bacillus BioBrick Box: generation and evaluation of essential genetic building blocks for standardized work with *Bacillus subtilis*. *Journal of biological engineering* 7, 29; 10.1186/1754-1611-7-29 (2013).

[4] Popp, P. F., Dotzler, M., Radeck, J., Bartels, J. & Mascher, T. The Bacillus BioBrick Box 2.0: expanding the genetic toolbox for the standardized work with *Bacillus subtilis*. *Scientific reports* 7, 15058; 10.1038/s41598-017-15107-z (2017).

[5] Nicolas, P. *et al.* Condition-Dependent Transcriptome Reveals High-Level Regulatory Architecture in *Bacillus subtilis*. *Science* 335, 1103–1106; 10.1126/science.1206848 (2012).

[6] Dérozier, S., Nicolas, P., Mäder, U. & Guérin, C. Genoscapist: Online Exploration of Quantitative Profiles Along Genomes via Interactively Customized Graphical Representations. *Bioinformatics* 37, 2747–2749; 10.1093/bioinformatics/btab079 (2021).

[7] Kagaya, Y. *et al.* NuFold: End-to-End Approach for RNA Tertiary Structure Prediction With Flexible Nucleobase Center Representation. *Nature Communications* 16; 10.1038/s41467-025-56261-7 (2025).

[8] Abramson, J. *et al.* Accurate Structure Prediction of Biomolecular Interactions with AlphaFold 3. *Nature* 630, 493–500; 10.1038/s41586-024-07487-w (2024).

[9] Wright, P. R. *et al.* CopraRNA and IntaRNA: Predicting Small RNA Targets, Networks and Interaction Domains. *Nucleic Acids Research* 42, W119-W123; 10.1093/nar/gku359 (2014).

[10] Mann, M., Wright, P. R. & Backofen, R. IntaRNA 2.0: Enhanced and Customizable Prediction of RNA–RNA Interactions. *Nucleic Acids Research* 45, W435-W439; 10.1093/nar/gkx279 (2017).

[11] Busch, A., Richter, A. S. & Backofen, R. IntaRNA: Efficient Prediction of Bacterial sRNA Targets Incorporating Target Site Accessibility and Seed Regions. *Bioinformatics* 24, 2849–2856; 10.1093/bioinformatics/btn544 (2008).
